# Low-grade appendiceal mucinous neoplasms with bowel obstruction

**DOI:** 10.1515/pp-2019-0020

**Published:** 2019-10-11

**Authors:** Constance Houlzé-Laroye, Clarisse Eveno

**Affiliations:** Department of Digestive and Oncologic Surgery, Claude Huriez University Hospital, Centre Hospitalier Universitaire (CHU) Lille, Université de Lille, Lille, France; INSERM Unité Mixte de Recherche 1172-JPARC Jean-Pierre Aubert Research Center, Team “Mucins, epithelial differentiation, and carcinogenesis”, Lille, France

**Keywords:** ileo-caecal intussusception, low-grade appendiceal mucinous neoplasma, mucin

## Abstract

**Background:**

Perforation of low-grade appendiceal mucinous neoplasms (LAMN) is characterized by its potential to spread mucin into peritoneal cavity, giving rise to pseudomyxoma peritonei, commonly treated with cytoreductive surgery and hyperthermic intraperitoneal chemotherapy.

Symptoms of intestinal obstruction and appendiceal infection are rare at early stages of the disease, while abdominal distension are observed in the later stages due to mucin spread.

**Methods:**

We report herein a case of LAMN with atypical symptoms in a 35-year-old woman with abdominal symptoms evoking an intestinal obstruction.

**Results:**

An abdominal CT scan revealed an ileo-caecal intussusception. An exploratory laparotomy and examination of the peritoneal cavity ruled out an exteriorization of mucin and the bowel was resected.

**Conclusions:**

The pathology analysis confirmed the diagnostic of LAMN. This report aims to raise awareness among surgeons, of rare clinical presentations that LAMN may show, to adapt the surgical treatment to these patients and assign them to referral centers.

We report a case of 35-year-old female patient with no medical history presented to the emergency department with acute vomiting and abdominal pain. The abdomen was distended with significant guarding in the left upper quadrant. An abdominal CT scan revealed an ileo-caecal intussusception through the left transverse colon (Figure 1, panels A, B with a target sign, arrow) caused by an appendicular mucocele filled with mucin, presenting parietal calcification (Figure 1, panel C, arrow). An exploratory laparotomy and examination of the peritoneal cavity ruled out an exteriorization of mucin and the bowel was resected. The pathology analysis confirmed the low-grade appendiceal mucinous neoplasms (LAMN) diagnosis with necrosis of appendiceal basis [1]. A perforation of LAMN is characterized by its potential to spread mucin into peritoneal cavity, giving rise to pseudomyxoma peritonei, commonly treated with cytoreductive surgery and hyperthermic intraperitoneal chemotherapy [2, 3]. Symptoms of obstruction and appendiceal infection at early stages of the disease, while abdominal distension is observed in the later stages due to mucin spread.

**Figure 1:**
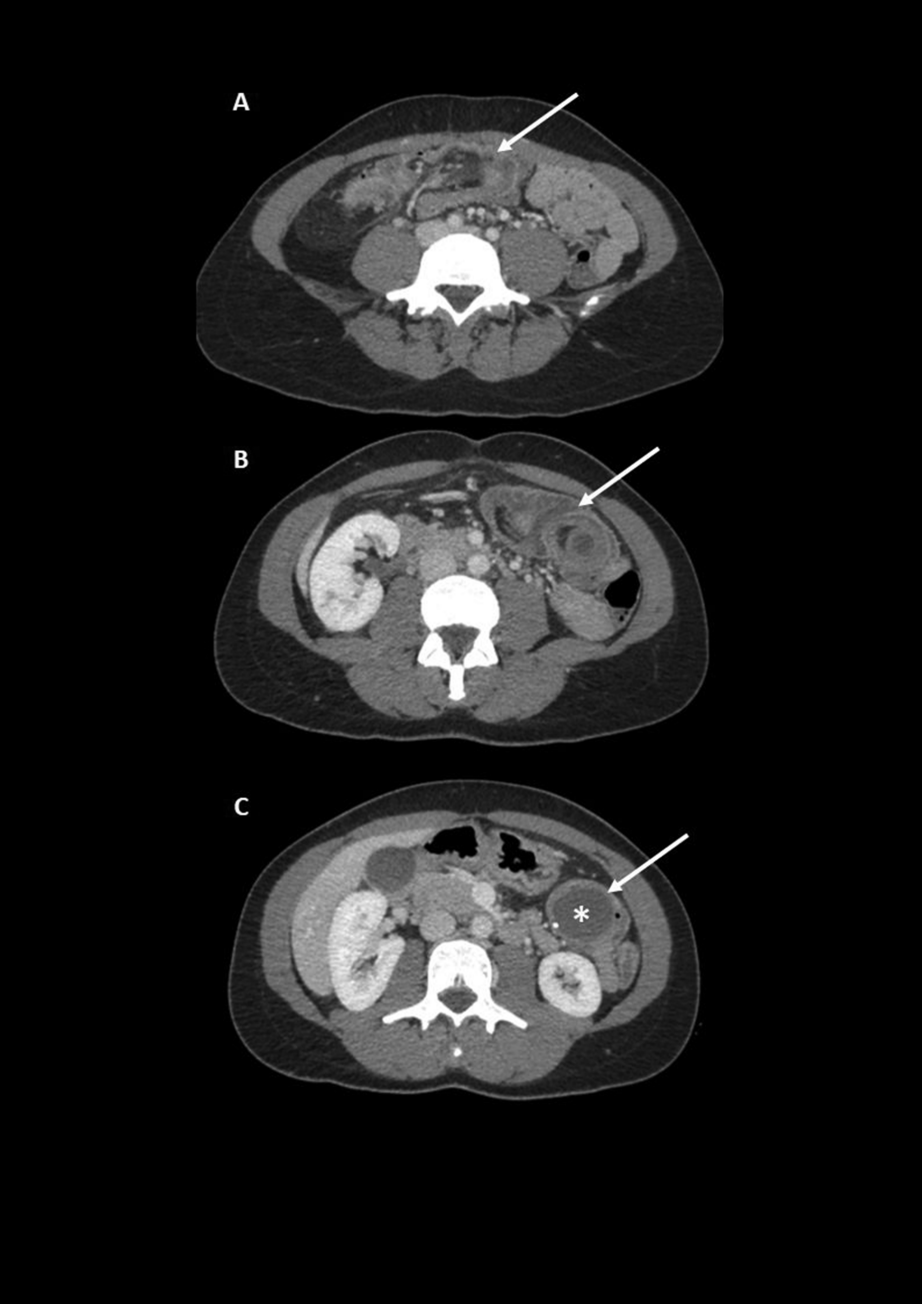
Abdominal CT scan revealed an ileo-caecal intussusception through the left transverse colon (panels A, B with a target sign, arrow) caused by an appendicular mucocele filled with mucin, presenting parietal calcification (panel C, arrow).
